# Approaching the physical limits of specific absorption rate for synthetic antiferromagnetic nanodisks in hyperthermia applications

**DOI:** 10.1039/d5bm00739a

**Published:** 2025-09-09

**Authors:** S. Scheibler, H. Wei, J. Ackers, S. Helbig, S. Koraltan, R. Peremadathil-Pradeep, M. Krupinski, M. Graeser, D. Suess, I. K. Herrmann, H. J. Hug

**Affiliations:** a Magnetic & Functional Thin Films Laboratory, Empa, Swiss Federal Laboratories for Materials Science and Technology Ueberlandstrasse 129 8600 Dübendorf Switzerland hans-josef.hug@empa.ch ingeh@ethz.ch dieter.suess@univie.ac.at; b Nanoparticle Systems Engineering Laboratory, Institute of Energy and Process Engineering (IEPE), Department of Mechanical and Process Engineering (D-MAVT), ETH Zurich Sonneggstrasse 3 8092 Zurich Switzerland; c Nanomaterials in Health Laboratory, Empa, Swiss Federal Laboratories for Materials Science and Technology Lerchenfeldstrasse 5, 9014 St. Gallen Switzerland; d Fraunhofer IMTE, Fraunhofer Research Institution for Individualized and Cell-Based Medical Engineering Mönkhofer Weg 239a 23562 Lübeck Germany; e Physics of Functional Materials, Faculty of Physics, University of Vienna Kolingasse 14-19 1090 Vienna Austria; f Research Platform MMM Mathematics – Magnetism – Materials, University of Vienna Kolingasse 14-19 1090 Vienna Austria; g Physics of Functional Materials, Faculty of Physic, University of Vienna Kolingasse 14-19 1090 Vienna Austria; h Department of Physics, University of Basel Klingelbergstrasse 82 4056 Basel Switzerland; i Institute of Nuclear Physics Polish Academy of Sciences Radzikowskiego 152 31-342 Kraków Poland; j Chair of Metrology, University Rostock Albert-Einstein-Str. 2 18059 Rostock Germany; k Ingenuity Lab, Balgrist University Hospital and University of Zurich Forchstrasse 340 8008 Zürich Switzerland inge.herrmann@uzh.ch ingeh@ethz.ch

## Abstract

Magnetic nanoparticle-based hyperthermia presents a promising approach to treating malignant solid tumors that are resistant to conventional therapies such as chemotherapy and radiation. However, the therapeutic potential of superparamagnetic iron oxide nanoparticles (SPIONs) is limited by low saturation magnetization, superparamagnetic behavior, and broad particle size distribution. Here, we present synthetic antiferromagnetic disk particles (SAF-MDPs) designed through micromagnetic modeling to maximize hysteretic heating while maintaining suspension stability. The SAF-MDPs feature in-plane magnetization optimized *via* uniaxial anisotropy adjustments to prevent spin-flop phenomena and eliminate hysteresis-free loops along the hard axis. Mechanofluidic modeling was used to assess particle alignment under an alternating magnetic field (AMF), and advanced magnetic characterization, including in-vacuum single-particle magnetic force microscopy, was employed to elucidate the switching process between antiferromagnetic and ferromagnetic states. The resulting SAF-MDPs approach the theoretical maximum specific loss power (SLP) allowed under the biological discomfort level, yielding significantly higher heating efficiency than SPIONs. This combined modeling–fabrication–characterization strategy opens a pathway toward magnetic hyperthermia agents operating near fundamental performance limits, with potential for further optimization through material choice and coupling-engineering strategies.

## Abstract 2

Antiferromagnetic disk particles with optimized anisotropy achieve near-maximal heating efficiency under safe alternating magnetic field exposure, surpassing iron oxide nanoparticles and advancing magnetic hyperthermia therapy.

## Graphical abstract

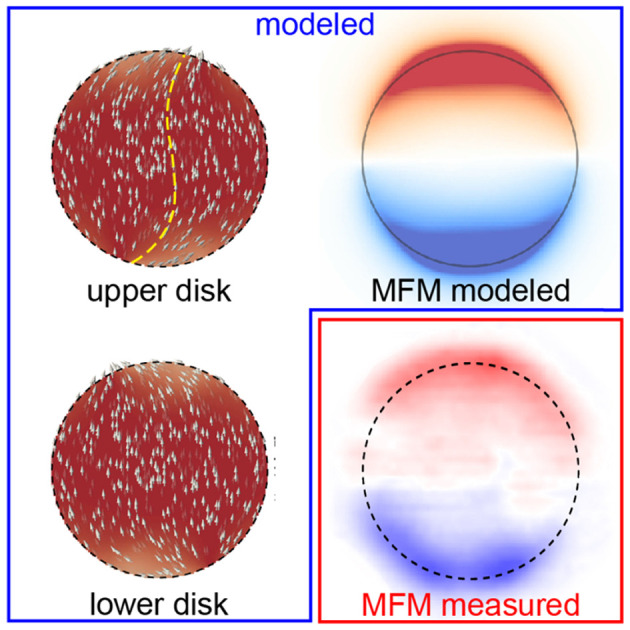

Magnetic hyperthermia emerges as a promising approach for the elimination of cancerous tissues. While it is generally regarded as a valuable addition to established treatment options such as surgery, radiotherapy, and chemotherapy, magnetic hyperthermia in its current form faces considerable constraints due to poor heating efficiency and unfavorable heat dissipation. These limitations lead to drastic consequences, including the inability to eradicate small sized tumors. Additionally, the poor heating efficiencies of contemporary magnetic hyperthermia necessitate the delivery of large doses of nanoparticles to the tumors. This requirement further limits the range of tumors that can be effectively targeted and treated, as not all tumor sites can safely or feasibly receive such high concentrations of nanoparticles. Magnetic nanoparticle based hyperthermia typically employs superparamagnetic iron oxide nanoparticles (SPIONs). Such particles have been approved for the treatment of recurrent glioblastoma in Europe, and more recently, by the U.S. Food and Drug Administration (FDA) for clinical trials for the treatment of prostate and pancreatic cancer. Owing to the necessity for substantial doses of particles within the tumor tissue, these particles are commonly administered directly into the tumor, its surrounding vasculature, or the peritumoral area prior to the application of an alternating magnetic field (AMF). This leads to magnetic hysteresis losses within the particles and consequently to a locally increased temperature leading to tumor cell death. The SPIONs are typically manufactured bottom-up, by chemical synthesis, carefully tuning their size distribution such that the largest particles have a volume and with it an anisotropy energy barrier which can be overcome at room temperature within a short timescale to prevent agglomeration. This is to obtain superparamagnetic properties of the particles and with it a vanishing magnetic moment at zero applied field needed to minimize an attractive magnetic inter-particle interaction which would destabilize a particle suspension. For slowly alternating magnetic fields, for example, for the field sweeping time in a classical vibrating sample magnetometry (VSM) experiment, the *M*(*H*)-loop of superparamagnetic particles is Langevin-like and thus does not show any coercivity and hysteresis^1^ (blue curve in Fig. 1a).

**Fig. 1:**
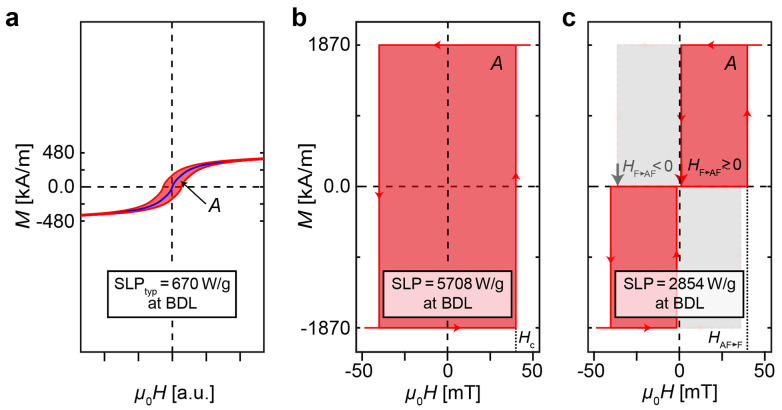
Physics-based limits of specific loss parameters under the boundary conditions of the biological discomfort level. (a) *M*(*H*)-loop of a superparamagnetic nanoparticle particle showing Langevin-like loop without hysteresis for slowly varying fields (blue curve) and developing a small hysteretic loss (red-shaded area) for fields oscillating at higher frequencies. (b) Ideal *M*(*H*)-loop of a ferromagnetic disk particle (F-MDP) leading to the largest hysteretic loss (red-shaded area) of 5708 W g_CoFe_^−1^ possible under consideration of the biological discomfort level. (c) Ideal *M*(*H*)-loop of a synthetic antiferromagnet (AF) disk particle (SAF-MDP) with *m*(*H* = 0) = 0 (red-shaded area). The maximum SLP is half the size of that of the F-MDP, but can be increased for a SAF-MDP with a reduced antiferromagnetic (AF) coupling (light gray shaded additional area). Then the magnetic moment does not longer vanish at zero field, and a demagnetization procedure has to be applied to re-set the SAF-MDP into its AF-coupled ground state.

However, because SPIONs exhibit effective magnetic anisotropy *K* due to crystalline and/or shape anisotropy *K*, the *M*(*H*)-loop develops a finite hysteretic loss area *A* (red curve and shaded area in Fig. 1a) increasing with the frequency *f* of the applied AMF. Although the size and anisotropy of the SPIONs can be optimized to increase the hysteretic loss area for the specific AFM frequencies applied in an hyperthermia application, the maximum obtainable loss remains fundamentally limited,^1,2^ as discussed in more detail in SI section S1.

It is further noteworthy that the product of the field amplitude *H* and frequency *f* remains limited because in a hyperthermia application, healthy body tissue not loaded with the magnetic particles may overheat due to Eddy current losses. Hergt and Dutz^3^ have reported a biological discomfort level (BDL) as *H* × *f* < 5 × 10^9^ Am^−1^ s^−1^. Slightly lower limits of 1.8 or 2 × 10^9^ Am^−1^ s^−1^, have been reported by Jordan *et al.*^4^ for the treatment of prostate tumor patients and Mamiya *et al.*^5^ considering the cooling by the blood flow. Here we will use the BDL by Hergt and Dutz.^3^ We strongly emphasize that such a BDL should be considered when comparing the heating efficacy of different SPNPs, which is unfortunately often ignored or not even stated in many previous works. With this in mind one may for example consult the table of the specific loss power (W g^−1^ magnetic material) (SLP) values given in Gavilán *et al.*,^1^ which, considering this limit, is in good agreement with the maximum SLP estimated by Ruta *et al.*^2^ for SPIONs.

Compared to chemical synthesis, top-down fabrication of particles offers a large variety of design options in view of the choice of materials, magnetic properties, but also concerning the particle size and shape which can be defined by typical micro-fabrication approaches. Layers of magnetic materials, along with additional layers, can be employed for various purposes, such as achieving optimized growth conditions, enhancing interfacial anisotropies, or facilitating magnetic interlayer coupling. Additionally, sacrificial layers may be utilized to enable the subsequent separation of disk-shaped islands from the substrate. These layers and architectures thereof play a critical role in tailoring the physical and magnetic properties of the structures for specific applications. In addition, further layers may be introduced for oxidation protection or for facilitating a successive (bio)chemical functionalization. To achieve stable suspensions of such disk-shaped particles, the total magnetic moment at zero field must vanish. This can be accomplished with synthetic antiferromagnet disk particles (SAF-MDP) consisting of two ferromagnetic layers (FL) that are antiferromagnetically (AF) coupled. The AF-coupling between the two FLs can be obtained either through the stray fields of the two F layers^6^ for the case of SAF-MDP with in-plane magnetization or by an antiferromagnetic Ruderman–Kittel–Kasuya–Yosida (RKKY) interaction,^7–9^ for example occurring by a 0.6 nm-thick Ru interlayer, as typically employed for SAF-MDP with perpendicular magnetization. Despite these approaches, hysteretic losses have remained nearly absent,^6,10^ or the fields required to switch MDPs into ferromagnetic alignment have been too high^11^ for practical hyperthermia applications (see SI section S2 for further information). Consequently, recent research has redirected focus toward using SAF-MDPs for the magnetomechanical destruction of cancer cells instead.^12,13^

In our work, we establish the fundamental limits for the maximum achievable hysteretic losses and employ micromagnetic and fluid-mechanical modeling together with single particle micromagnetic characterization to obtain SAF-MDPs with maximized hysteretic losses opening an avenue to reach the absolute limits of the heating power given by physics.

## Results and discussion

### Maximizing hysteretic losses for in-plane SAF-MDPs

The hysteretic loss of any system is fundamentally limited by the material with the highest saturation magnetization in Nature, which for the case of magnetic CoFe thin films is *M*_s_ = 1870 kA m^−1^.^14^ For a perfectly rectangular hysteresis loop (Fig. 1b) a SLP_F,max_ = 4*μ*_0_ × *M*_s_(*H*_c_ × *f*)/*ρ*_CoFe_ = 5708 W g_CoFe_^−1^ would then be obtained (here *ρ*_CoFe_ = 8233 kg m^−3^ was used). Note that this is the highest SLP possible by the limits of Nature under the boundary condition of the BDL as given by Hergt and Dutz^3^ and that for a system reaching its saturation magnetization, the SLP is independent of the size of the coercive field. This is because within the boundaries of the BDL a larger *H*_c_ leads to a correspondingly smaller *f*.

The maximum SLP for superparamagnetic magnetite particle (SPIONs) with *M*_s_ = 480 kA m^−1^ must hence be much smaller than 1747 W g_Fe_3_O_4__^−1^ or 2413 W g_Fe_^−1^ which would be obtained only for a perfectly rectangular *M*(*H*)-loop. However, because magnetic nanoparticles with a finite anisotropy are only superparamagnetic if the energy barrier between the two magnetic states can be overcome by the thermal energy available at room temperature, the switching field distribution of an ensemble of superpamagnetic particles for an applied AMF is inherently wide even for mono-disperse particles, and thus always yields a non-rectangular hysteresis loop^15^ (see also SI, section S1). Ota *et al.* measured the imaginary part of the susceptibility of Synomag-D dispersed in diluted water and extracted from it the specific loss power (SLP) under BDL. They obtained SLP = 75 W per g-Fe (*H*_0_ = 16 kA m^−1^, *f* = 314 kHz) and to SLP = 62 W per g-Fe (*H*_0_ = 4 kA m^−1^, *f* = 1260 kHz)^16^

Additionally, due to thermal activation, saturation magnetization cannot be achieved in AC field cycles. For example, Co-like superparamagnets with diameters of 13 nm, subjected to an AC field with amplitude of 5 mT and a frequency of 100 kHz, only reach a magnetization of about 50% of the saturation magnetization *M*_s_.^2^ As shown in^2^ the obtained hysteresis loops are elliptical like, which can not utilize the entire area of the rectangle with area 4*μ*_0_*H*_c_*M*_s_.

We argue that an anisotropy energy substantially exceeding thermal energy and thus a sufficiently large particle volume is a necessary but not yet sufficient condition to obtain the desired rectangular hysteresis loop. To ensure suspension stability, it is further essential that the particle possesses a zero net magnetic moment in the absence of an external field or at least can be reset into a zero moment ground state. These conditions can in principle be satisfied by a synthetic antiferromagnet disk particle (SAF-MDP) consisting of two antiferromagnetically (AF) coupled CoFe FLs with equal magnetic moments exhibiting a hypothetical hysteresis loop as depicted by the red shaded area in Fig. 1c.

For fields *H* ≥ 0, the ideal *M*(*H*)-loop of such a SAF-MDP is defined by a transition at a field *H* > *H*_AF→F_, where the FLs switch from the AF-coupled ground state to a ferromagnetic (F) alignment, and a return to the AF ground state at a field *H*_F→AF_ ≳ 0. In this configuration, the maximum energy absorption rate of an ideal SAF-MDP is 2854 W g_CoFe_^−1^ (red shaded area in Fig. 1c), which is half of the rate achievable by a purely ferromagnetic disk particle.

By reducing the AF coupling strength, SAF-MDPs with increased hysteretic losses approaching those of a ferromagnetic particle (represented by the light gray shaded area in Fig. 1c) can be engineered. Suspension stability can still be obtained, as the magnetic ground state still exhibits an AF alignment and can be achieved through appropriate demagnetization techniques.

### SAF MDP system design and fabrication

The micromagnetic state of small disk-shaped magnetic particles is governed by various parameters, including the disk diameter, thickness, and the intrinsic properties of the magnetic layer such as saturation magnetization, exchange stiffness, and magnetic anisotropy. Typically, small disks with a thin, single FL form a uniform mono-domain state, as documented by Cowburn *et al.*^17^ In contrast, larger disks tend to exhibit multi-domain or vortex states, as noted by Usov *et al.*^18^ and Guslienko *et al.*^19^ Disks with intermediate sizes are expected to maintain a mono-domain state, albeit with magnetic moment directions locally deviating from the easy in-plane axis.

To achieve a hysteretic easy-axis magnetization process with a low switching field *H*_AF→F_ compatible with the AMF amplitudes typical in hyperthermia experiments, along with a narrow switching field distribution and *H*_AF→F_ ≳ 0, we conducted extensive micromagnetic modeling. Our objective was to identify optimal system parameters for SAF-MDPs with a 500 nm diameter, resulting in the desired *M*(*H*)-loop even with modest parameter variations. We determined that magnetic layers with a saturation magnetization *M*_s_ ≈ 1350 kA m^−1^, an F-layer thickness of 6–7 nm, and a uniaxial magnetic anisotropy of *K*_u_ = 20 kJ m^−3^ meet these criteria (see SI section S3 for further details). Note that we also performed extensive micromagnetic modeling for disks with smaller diameters, revealing that identical *M*(*H*)-loops can be obtained if the magnetic layer thickness is scaled proportionally with the particle diameter. If the magnetic layer thickness is kept constant, the antiferromagnetic coupling—and consequently the switching field *H*_AF→F_ increases to levels inconvenient for the AMF amplitudes typically available in hyperthermia setups. Moreover, the field *H*_F→AF_ also shifts away from *H*_F→AF_ ≳ 0, thereby reducing the SLP under the boundary conditions of the biological discomfort level.^3^ Such an undesirably large antiferromagnetic coupling could, however, be reduced by introducing an intermediate layer providing a Ruderman–Kittel–Kasuya–Yosida (RKKY) interlayer exchange, for example by replacing the AlZr interlayer with a Pt interlayer.

**Fig. 2:**
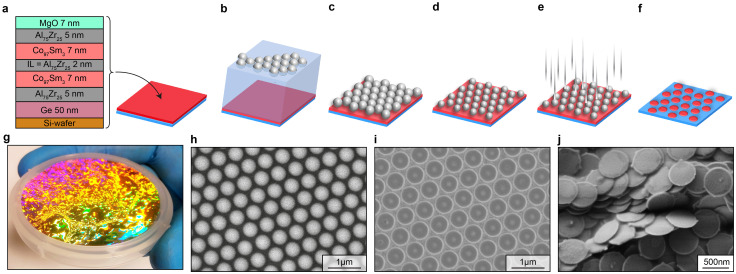
SAF MDP design and fabrication. (a) Multilayer system containing two 7 nm thick Co_97_Sm_3_ layers separated by a 2 nm-thick Al_75_Zr_25_ layer covered by 5 nm Al_75_Zr_25_ for oxidation protection was deposited onto an 5 nm Al_75_Zr_25_ on a 50 nm-thick sacrificial Ge layer on a 2 inch silicon wafer substrate. (b)–(f) Schematics of the nanopatterning process employed in our work to fabricate the SAF-MDP islands still attached to the wafer substrate (f). (g) Wafer with the SAF-MDP multilayer structure and self-assembled polystyrene (PS) sphere as schematically depicted in (c). (h) Scanning electron microscopy (SEM) image of the self-assembled PS spheres with a diameter of 500 nm reduced from the initial diameter of 660 nm by oxygen plasma etching. (i) and (j) SEM pictures of the SAF-MDP attached to the wafer and re-deposited onto a substrate from an SAF-MDP suspension, respectively.

While SAF-MDP with large perpendicular anisotropies can be obtained by interfacial anisotropies occurring for example in Co/Pt multilayers,^20^ achieving a large uniaxial anisotropy in the order of 20 kJ m^−3^ in in-plane magnetized systems is more challenging. Here we use amorphous Co_1−*x*_Sm_*x*_ layers sputter-deposited in an applied in-plane magnetic field, for which Magnus *et al.*^21^ have demonstrated giant in-plane anisotropies up to about 200 kJ m^−3^ for Sm contents of 22%. For the 20 kJ m^−3^ required according to our micromagnetic modeling work, much smaller concentrations of Sm were tested and an optimal concentration of 3% was determined for film with thickness between 6 and 8 nm deposited onto 5 nm-thick Al_75_Zr_25_ seed layers to promote an amorphous growth. At the interfaces magnetic layers with reduced magnetic moments were observed which accounted for a total magnetic dead layer thickness of 0.7 nm (see SI section S3). To obtain the magnetic layer thickness of 6.3 nm used for our micromagnetic modeling work, a SAF structure comprised of two 7 nm thick Co_97_Sm_3_ layers separated by a 2 nm-thick Al_75_Zr_25_ layer covered by 5 nm Al_75_Zr_25_ for oxidation protection was deposited onto an 5 nm Al_75_Zr_25_ seed (see SI section S4). As a substrate an 2 inch silicon wafer with an additional 50 nm-thick Ge layer was used (Fig. 2a). We note that leaching of Co from the CoSm ferromagnetic layers could lead to undesirably high cytotoxicity. Potential mitigation strategies include replacing the CoSm layers with less toxic magnetic materials such as polycrystalline Fe, amorphous FeB, or Fe–Sm alloys, where the required in-plane anisotropy could potentially be achieved by oblique sputter deposition^22^ or, as in the present work, by sputtering in an applied magnetic field. In this study, we nevertheless employed the well-established CoSm alloy system to ensure reproducible magnetic properties and to match the anisotropy parameters predicted by our micromagnetic modeling.

For the patterning a polystyrene-based lithography approach adapted from Giersig *et al.*^23^ was employed with additional process improvements introduced by other authors^24–26^ (Fig. 2b–j). This approach offers high scalability to the size of several in^2^ and ensures good repeatability at minimal cost since no special equipment is required. For this, polystyrene (PS) beads prepared *via* free radical initiated polymerization with a diameter of 660 nm were self-assembled at the water–air interface, creating a highly ordered hexagonally packed monolayer of PS submicron spheres (Fig. 2g).^25,27,28^ The quality of the self-assembled periodic pattern of PS beads over a full 2-inch Si wafer was confirmed through visual inspection (Fig. 2g). Following oxygen plasma reactive ion etching to separate the PS spheres and reach a bead diameter of 500 nm (Fig. 2d and h), Ar milling was performed to pattern the wafer (Fig. 2e). Afterwards the PS beads were removed by ultrasound in water, yielding circular disk-shaped pillars still attached to the wafer (Fig. 2f and i). The height of the MDPs on the wafer was determined as approximately 50 nm and is higher than the total thickness of the magnetic multilayer (33 nm). This is because the ion-milling process was continued into the Ge sacrificial layer to ensure that the bottom AlZr layer has also been removed. Finally, the MgO top sacrificial layer was removed using citric acid and the Ge bottom sacrificial layer was dissolved in a final step to release the SAF MDPs from the wafer into suspension. Fig. 2j then shows the particles re-deposited from the suspension onto a wafer surface for successive scanning electron microscopy observation.

### Magnetic and hyperthermia characterization and extended micromagnetic and mechanofluidic modeling

**Fig. 3:**
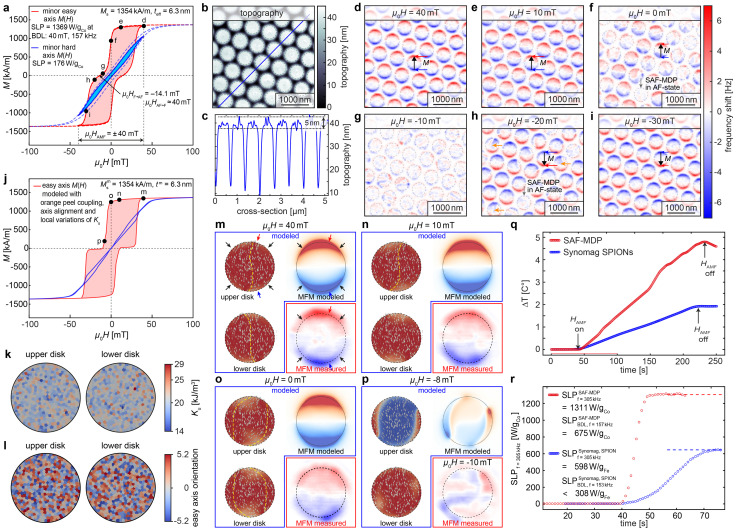
SAF-MDP magnetic and hyperthermia characterization. (a) VSM measurements of the easy axis *M*(*H*)-loop (solid and dashed red curves, for the minor and major loop, respectively), hard axis *M*(*H*)-loop (solid and dashed blue curves, for the minor and major loop, respectively) of our SAF-MDPs still attached to the wafer (*D* = 500 nm, 7 nm-thick Co_97_Sm_3_ F layers separated by 2 nm of Al_70_Zr_30_). The labels (d)–(i) Refer to the corresponding MFM data displayed in panels (d)–(i). (b) Topography image and (c) cross-section of the SAF-MDP islands. (d)–(i) Magnetization reversal of the SAF-MDP studied by magnetic force microscopy (MFM) performed in fields applied along the easy axis, ranging from 40 mT to −30 mT. (j) Easy and hard axis (red and blue lines, respectively) *M*(*H*)-loops obtained from a refined micromagnetic model using distinct distributions anisotropies and easy axes alignments of the grains in the upper and lower disks as displayed in (k) and (l), respectively, and employing a roughness-induced ferromagnetic orange-peel coupling between the FLs. (m)–(p) Show magnetic moment distributions of the upper and lower disk of a SAF MDP (top left and bottom left), MFM patterns calculated from these (top right) and experimental MFM data (bottom right) for 40, 10, 0, and −8 mT applied fields. (q) Temperature rise as a function of time for suspensions of 80 μg_Co,Fe_ ml^−1^ of SAF-MDP and Synomag superparamagnetic nanoparticles. (r) Evolution of the SLP values with time for suspensions of the AF-MDP and Synomag superparamagnetic nanoparticles calculated from (q).

After fabrication, the SAF-MDPs were characterized, and Fig. 3a displays the easy-axis *M*(*H*)-loop (solid and dashed red curves for the minor and major loops, respectively) alongside the hard-axis *M*(*H*)-loop (solid and dashed blue curves for the minor and major loops, respectively) for the SAF-MDPs still attached to the wafer, as measured by VSM. The easy-axis field required to align the SAF-MDPs into the F-state is approximately 40 mT, closely matching the modeled loop (SI Fig. S1a, red curve), while switching from the F- to the AF-aligned state requires a small negative field (after saturation in a positive field). Both switching processes occur over a slightly broader field range, suggesting the presence of complex domain states during switching.

The hysteretic losses calculated from the minor easy and hard axis (solid red curve/red area and solid blue curve/blue area in Fig. 3a, respectively.) *M*(*H*)-loops for a *±B* = 40 mT field excursion are 77.6 and 9.97 kJ m^−3^, respectively. From these hysteretic losses, SLP values of 1369 and 176 W g_Co_^−1^ are obtained at the BDL for *B* = 40 mT, *f* = 157 kHz (see eqn (S3) of the SI section S1).

Hence, the easy axis SLP expected from the *M*(*H*)-loop is only about 0.48 of the 2854 W g_Co_^−1^ calculated for an ideal SAF-MDP (Fig. 1c). A reduction of the SLP by a factor of 0.724 can be expected from the saturation magnetization of 1354 kA m^−1^ of the CoSm ferromagnetic layer used here, which is considerably smaller than the 1870 kA m^−1^ of CoFe alloy films.^14^ Hence, to obtain the observed total SLP reduction, the remaining factor of 0.662 must be attributed to the more gradual switching from the AF to the F-state. The latter demonstrates the importance of sharp switching between the AF and F-states.

To gain insight in the nature of the switching process, we employed a home-built magnetic force microscope working under vacuum conditions to obtain increased measurement sensitivity.^29,30^ Advanced techniques to disentangle contrast contributions from the topography (Fig. 3b, cross-section c) from those arising from the magnetic field and deconvolution techniques^29,30^ were employed to obtain the frequency shift data depicted in Fig. 3d–i for different fields applied along the in-plane easy axis of the SAF-MDPs. Figure panels 3d and e show the SAF-MDPs micromagnetic state at saturation at *μ*_0_*H* = 40 mT and at *μ*_0_*H* = 10 mT. Half-moon shaped red and blue features are apparent at the upper and lower edges of the SAF-MDPs (red and blue arrows in Fig. 3d and e). The magnetic force microscopy (MFM) results obtained at zero field (Fig. 3f) show that some of the SAF-MDPs have switched back into their AF ground state but several disks still show a substantial red/blue MFM contrast, indicating an incomplete switching process. The latter is also apparent from the magnetic remanence (point f in Fig. 3a). At a field *μ*_0_*H* = −10 mT the magnetization nearly vanishes (point g in Fig. 3a). The MFM image (Fig. 3g) consequently shows on a very small granular contrast indicating the magnetic moments in the upper and lower disks are essentially antiparallel. Hence the stray fields generated by the opposite poles of the two disks essentially compensate each other. At −20 mT many disks have already switched into their F-states with a south pole (blue half moon) at the top and a north pole (red half moon) at the bottom (Fig. 3h), whereas at −30 mT all disks visible in the MFM image (Fig. 3i) are in the F state, compatible with the *M*(*H*)-loop which is almost saturated at point i.

To complement the initial micromagnetic modeling, which assumed ideal magnetic layers with homogeneous magnetic properties and was used to identify an optimal parameter set for fabrication, we performed advanced simulations to gain a deeper understanding of the parameters influencing SAF-MDP switching behavior in realistic material systems. In these refined models, the SAF-MDPs were represented with a granular microstructure, incorporating 20 nm-diameter grains with a Gaussian distribution of anisotropy values centered at *K*_u_ = 20 kJ m^−3^ and a standard deviation of ±2 kJ m^−3^, combined with a uniform variation in anisotropy axis orientation of ±3° (Fig. 3k and l). Interlayer coupling effects (arising from orange peel coupling) were also included. These features led to moderate domain-wall pinning, which accounts for the observed minor particle-to-particle switching differences (Fig. 3d–i).

However, the experimental shift in field required for the SAF-MDPs to revert to their AF ground states after saturation is not yet reproduced. Instead, a reduction in AF coupling between the ferromagnetic layers (FLs) is required. A likely source of this reduction is ferromagnetic “orange-peel” coupling arising from interlayer roughness.^31^ To replicate this effect in the model, an orange-peel exchange coupling constant *J*_op_ = −*t*^eff^_FL_*μ*_0_*M*^eff^_s_|*H*_shift_|/2 = −0.032 mJ m^−2^ was applied. This adjustment enabled close alignment between the modeled (Fig. 3j) and experimentally observed (Fig. 3a) easy-axis *M*(*H*)-loops. The resulting shift *μ*_0_|*H*_shift_| ∼ 15 mT reflects the difference in loop centers with and without orange-peel coupling, providing insight into the interplay of anisotropy and coupling effects in SAF-MDP switching.

Fig. 3m–p then display the modeled magnetic moment distributions of the top and bottom disks (left side, top and bottom images) together with a comparison of the simulated and measured MFM images (right top and bottom images) for different applied fields. For this, specific SAF-MDP were selected for each field to achieve a reasonable match with the modeled MFM data. We note that this is justified as the real defect distribution in a specific SAF-MDP island remains unknown, and hence only a qualitative agreement can be expected. For a field of 40 mT both FLs are almost saturated, and the magnetic moments are well aligned to the direction of the anisotropy axis direction (yellow dashed curves) with a slight s-shaped orientational deviation from the easy axis. The red and blue half moons (Fig. 3m, MFM model and measurement) arise from the positive and negative magnetic charges generated by the divergence of the magnetization field at the upper and lower disk boundaries, respectively. Note that the divergence is strongest if the local orientation of the magnetic moment vector is perpendicular to the disk boundary (at the top and bottom centers, red and blue arrows) and weaker at the disk sides (black arrows). When the applied field is reduced, the stray field of one disk acting on the moments of the other disk leads to a gradual deviation of the magnetic moments away from the easy magnetization axis at 10 and 0 mT (see increased curvatures of the dashed yellow lines in Fig. 3n and o, upper and lower disks) and finally to the formation of reversal domains at −8 mT (Fig. 3p) and consequently to a low moment magnetic state close to the ideal AF-oriented ground state. The dotty features visible for some of the SAF-MDPs (Fig. 3n arise from local divergences of the magnetization field). Compatible with the experimental *M*(*H*)-loop, at −10 mT, the SAF-MDP has switched back almost perfectly into its AF-aligned ground state (Fig. 3p), here (modeled at −8 mT) with the magnetic moments of the upper disk mainly pointing point down (blue color) apart from a small and narrow domain at the right side, and the lower disk pointing up (red color). The simulated MFM contrast then is very weak agreeing well with that observed in the experiment.

For the measurement of the specific loss parameter, an LCC resonance circuit with impedance matching permitting the application of ac-fields (AMF) with an amplitude up to 50 mT at its resonance frequency of 305 kHz was developed (see supporting materials for a more detailed description).

For the quantification of the SLP of our SAF-MDPs *in vivo*-relevant settings, SAF-MDPs were harvested from two 2-inch wafers (Fig. 2l) fully covered with hexagonal patterns of SAF-MDP islands (Fig. 2n). The SAF-MDP islands have been removed from the wafer substrate by dissolution of the Ge sacrificial layer in 35% H_2_O_2_, collected by a permanent magnet, while the aqueous phase was replaced by deionized water four times to finally achieve a suspension of SAF-MDPs. We find a yield per wafer of 134 μg of SAF-MDP mass, equivalent to 66% of the theoretically possible maximum, assuming a perfect close-packed hexagonal pattern of SAF-MDP islands over the entire wafer and a complete recovery of all particles. For the SLP measurements, a suspension of 1 ml with 134 μg of SAF-MDP consisting of a concentration of 80 μg_Co_ ml^−1^ was used. The temperature of the suspension was adjusted to match the 20 °C of the coolant liquid used for the coil of the SLP apparatus to prevent a parasitic heat loss. Then, an AMF of 40 mT and 305 kHz was applied. The obtained time dependence of the temperature rise of the SAF-MDP suspension is plotted in Fig. 3q (red circles) together with that obtained for a suspension of Synomag^32,33^ SPIONs with an Fe concentration of also 80 μg ml^−1^ (blue circles). Clearly, the temperature rise of the SAF-MDP is noticeably faster than that of the Synomag SPION particles. The SLP as a function of the first 60 s, *i.e.* 20 s beyond the time where the AMF was turned on is plotted in Fig. 3r, again for the SAF-MPD (red circles) and the Synomag SPIONS (blue circles). For the SAF-MDP a SLP of 1311 W g_Co_^−1^ was obtained while that of the SP was only 598 W g_Fe_^−1^. However, note that the 40 mT AMF applied at a frequency of 305 kHz results in a field-frequency product of 9.7 × 10^9^ Am^−1^ s^−1^ which is almost twice as large as the BDL. Consequently, at the BDL, for a frequency *f* = 157 kHz the SAF-MDP and Synomag particles would generate a SLP of about 675 W g_Co_^−1^ and <309 W g_Fe_^−1^, respectively. Note that for the SAF-MDP the scaling of the SLP is linear with the frequency, while for the superparamagnetic Synomag particles the area of the *M*(*H*)-loop shrinks at lower frequencies, leading to a further reduction of the SLP. The 675 W g_Co_^−1^ obtained for the SAF-MDP at the BDL is about 49.3% of the SLP obtained at the BDL from the area of the easy axis *M*(*H*)-loop measured by VSM (red area in Fig. 3a) which is 1369 W g_Co_^−1^ but about a factor of 7.8 larger than the SLP obtained from the hard axis *M*(*H*) loop measured by VSM (blue area in Fig. 3a which is 176 W g_Co_^−1^).

We note that the determined SLP is compatible with that obtained if about 41.8% of the SAF-MDP aligned their easy-axis and the rest of the particles aligned the hard-axis with the direction of the applied AMF. This differentiates our approach from earlier work, where either no^6,10^ or only very weak^20,34^ hysteretic losses were observed. For the case of the SAF-MDP with in-plane magnetic moments and without a well-define uniaxial anisotropy, the absence of the hysteretic loss was attributed to the occurrence of a spin-flop of the AF-coupled magnetic moments, followed by a hard-axis magnetization process.^6,10^ For the SAF-MDP with a strong perpendicular anisotropy,^20,34^ the SAF-MDP tended to align their hard axis with the AMF.

**Fig. 4:**
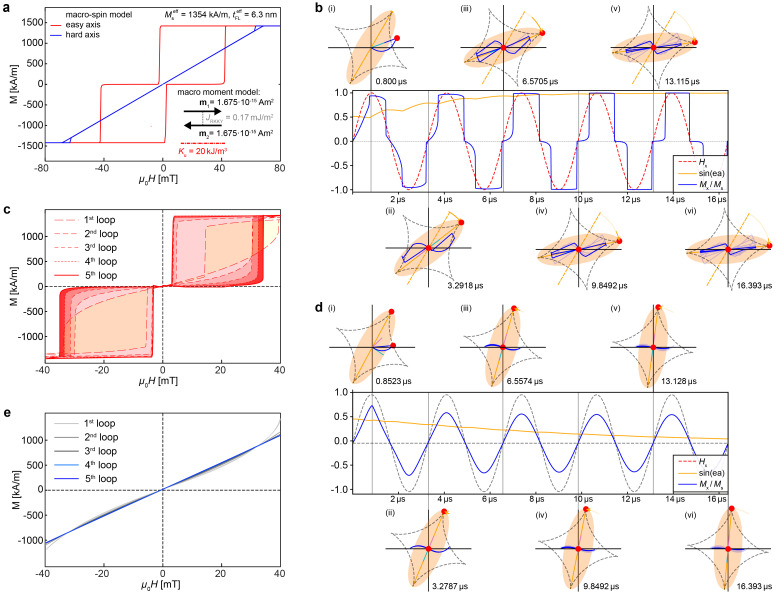
Mechanofluidic modeling of the SAF-MDP alignment to the AMF. (a) Easy (red curve) and hard axis (blue curve) *M*(*H*)-loops obtained from a simplified model comprised of two macroscopic magnetic moments **m**_1,2_ = 1.675 × 10^–15^ Am^2^ corresponding to the FL magnetic moment, AF coupled with *J*_RKKY_ = −0.085 mJ m^−2^ and experiencing a uniaxial anisotropy *K*_u_ = 20 kJ m^−3^. (b) Time evolution of the alignment of the particle's easy axis relative to the axis of the AMF (applied along the horizontal axis) for an initial angle of the easy axis of *ϑ* = 59°. The latter is below the critical angle for which the particle no longer aligns its easy axis to the AMF. The orange ellipses (i) to (vi) showcase the evolution of the particle's easy axis with the time of the applied AMF, while the small purple and turquoise lines inside the ellipses indicate the direction of the two magnetic moments **m**_1,2_ and the evolution of the total magnetic moment, respectively. The diagram between the upper three and lower three ellipses plots the amplitude of the AMF (dashed red curve), the evolution of the easy axis alignment to the AMF (orange curve), and the component of the total magnetic moment along the AMF axis (blue curve). (c) Evolution of the *M*(*H*)-loop and hysteresis. (d) Time evolution of the alignment of the particle's hard axis parallel to the axis of the AMF for an initial angle *ϑ* = 60° above the critical angle. (e) Decay of the hysteresis of the *M*(*H*)-loop with time as the particle aligns its hard axis to the AMF.

To elucidate the significant hysteretic losses observed in our experiments, which we attributed to an at least partial alignment of the easy axes of the SAF-MDP with the AMF, we conducted coupled micromagnetic and mechanofluidic modeling. For this, two AF-coupled macroscopic magnetic moments *m*_1,2_ = *M*_s_ × π*r*^2^*t*^*m*^ = 1.675 × 10^−15^ Am^2^, with *M*^eff^_s_ = 1354 kA m^−1^, *r* = 250 nm, and a ferromagnetic layer thickness *t*^eff^_FL_ = 6.3 nm were used. Further, a uniaxial anisotropy *K*_u_ = 20 kJ m^−3^, an AF-coupling constant^35^*J*_RKKY_ = −*t*^eff^_s_*μ*_0_*M*^eff^_s_|*H*_ex_|/2 = −0.085 mJ m^−2^, with *μ*_0_|*H*_ex_| = 20 mT and the viscosity of water *η* = 0.89 × 10^–3^ Pa s at a temperature *T* = 25 °C were employed. The viscosity used does not represent a tissue but rather the aqueous substance in which the experiments were carried out. The obtained easy axis *M*(*H*) loop (red curve in Fig. 4a) exhibits an abrupt switching between the AF and F states at fields comparable to those of the more gradual switching process observed in our experiment (see red *M*(*H*)-loop in Fig. 3a).

Our modeling then focused on a series of initial alignment angles between the easy axes of the SAF-MDP and the axis of the AMF with an amplitude of 40 mT and a frequency of 305 kHz, consistent with the parameters used in our experiments. We found that SAF-MDP with an initial easy-axis to AMF angle of 59°, align their easy axis with the AMF within a few oscillation cycles (Fig. 4b) leading to an increasing hysteretic loss finally approaching that of the easy axis process (see *M*(*H*)-curves one to five in Fig. 4c). For angles larger or equal to 60°, the SAF-MDP align their hard axis with the AMF (Fig. 4d) and, for the case of our model, the hysteretic losses vanish (see non-hysteretic *M*(*H*)-curves one to five in Fig. 4e). Our magneto-mechanofluid modeling thus reveals a critical angle of about 60°, and that about 66% of the SAF-MDP align their easy axis with the AMF and thus generate a hysteretic loss. This agrees reasonably well with our observation based on the experimental data which revealed that about 41.8% of SAF-MDPs aligned their easy axes with the AMF.

## Conclusions and perspectives

Our research investigated the potential of designer nanoparticles, optimized through micromagnetic modeling, to achieve maximum specific loss power (SLP) based on fundamental physical principles. This evaluation was conducted under the constraint of maintaining patient comfort, defined by the biological discomfort level established by Hergt and Dutz, which sets the permissible maximum product of frequency and field amplitude.^3^ The holistic design approach presented here enables micromagnetic optimization of the particle design, subsequent particle fabrication using a scalable microfabrication approach, followed by in-depth magnetic characterization using in-vacuum, high-sensitivity MFM gaining insights into the magnetic switching behaviour at a single particle scale. These in-depth characterization in tandem with micromagnetic and mechanofluidic modeling enable unprecedented insights into the particle behavior and reveals that the presence of a not yet perfect switching behavior between AF and F states as well as a yet partial alignment of the particle's easy axes with the AMF.

Our findings further suggest that for the applied AMF with an amplitude *μ*_0_*H* = ±40 mT, between 40–60% of the particles align their hard axis to the AMF and consequently do not contribute to the hysteretic loss. Further simulations however suggested that a full alignment of the particle's easy axis with the AMF is possible either by the application of dc-field alignment pulses or an initially increased ac-field amplitude.

This work provides comprehensive mechanistic insights into the design and behavior of magnetic particles, significantly advancing our understanding in this field. By elucidating the fundamental principles that govern particle performance, this work opens new avenues for the creation of particles whose SLP is restricted only by the inherent laws of physics. This in-depth understanding effectively removes the traditional constraints imposed by sub-optimal particle properties due to material selection and fabrication limitations, thereby unleashing the potential for more efficient magnetic particle designs. We emphasize that biocompatibility assessment is critical for translational relevance. Our recent *in vitro* studies on SAF-MDPs in human monocyte-derived macrophages demonstrated efficient uptake and cytotoxicity, consistent with reports on similar disk-shaped nanoparticles.^36^ While hemolysis testing represents an important next step, this lies beyond the current scope, which was focused on magnetic performance. Notably, the chosen amorphous Co_1−*x*_Sm_*x*_ system enabled the desired in-plane uniaxial anisotropy (*K*_u_ = 20 kJ m^−3^), yet comparable anisotropies may also be achievable with alternative, potentially less toxic materials such as polycrystalline Fe, amorphous FeB, or Fe_1−*x*_Sm_*x*_ with reduced Sm content. A systematic exploration of these alternatives, coupled with a rigorous quantitative analysis of their toxicity profiles, remains an important direction for future work. Ultimately, to establish clinical applicability, comprehensive *in vivo* experiments will be required to complement our current findings and validate both the safety and performance of SAF-MDPs under physiologically relevant conditions.

## Methods

### Magnetic multilayer deposition

The multilayers, including a 7 nm thick top sacrificial layer of MgO, were sputter-deposited onto a 2-inch Si wafer using an AJA DC/RF magnetron sputtering system under a working pressure of 10^−3^ Pa of Ar gas and a base pressure below 1 × 10^−8^ Pa. To establish a well-defined uniaxial in-plane magnetic anisotropy, the substrate was fixed to a custom-built sample holder containing two NdFe permanent magnets, generating a uniform field of 400 Oe within the substrate plane.

### Modeling

#### Micromagnetic simulations

The micromagnetic simulations were performed with the python library magnum.np^37^ using a finite difference method with a regular rectangular mesh. The real dimensions of the simulation box around the disk are 500 nm × 500 nm × 16 nm and is discretized with rectangular cells. Each layer is 6.3 nm thick and separated by a 3.4 nm thick spacer layer. This results in a simulation box of 100 × 100 × 3 cells, with a discretization of 5 nm × 5 nm × 6.3 nm, while the cells in the spacer layer have a size of 5 nm × 5 nm × 3.4 nm. The magnetic parameters in the spacer layer and the material outside of the disk radius are set to zero, *e.g. M*_s_ = 0. The micromagnetic framework solves the Landau–Lifshitz–Gilbert (LLG) equation^38–40^ in each cell, which describes the change of the magnetic moment according to all contributions to the effective field.1
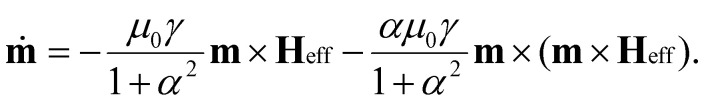


Here, *m* is the unit vector pointing in the direction of the magnetic moment, *μ*_0_ is the vacuum permeability constant, *γ* is the gyromagnetic ratio, *α* is the Gilbert damping parameter, and **H**_eff_ is the effective field. The first term describes the precession and the second term the damping of the magnetic moment. Each layer simulated with in-plane anisotropy, exchange coupling and also included is the demagnetization energy which is mainly responsible for the magnetic coupling of the two layers *via* the stray field. Additionally, Ruderman–Kittel–Kasuya–Yosida (RKKY) interactions are included to emulate the orange-peel effect by introducing a small alignment field. Lastly, an external field is applied in the simulations. This results in an effective field consisting of the anisotropy field, the exchange field in each layer, the demagnetization field, the RKKY field and the externally applied field. In order to avoid any numerical instabilities due to symmetry, the field is applied at an angle of 5 degrees relative to the easy axis and at an angle of 89 degrees for the hard axis simulations. The saturation magnetization in one layer is increased by 2% to further lower the symmetry of the system. For the simulations of the *M*(*H*)-loops (Fig. 3j) and the magnetic moment distributions and MFM data (Fig. 3m–p) the applied field was cycled from −100 mT to 100 mT changing by 2 mT each 1 × 10^–9^ and finishing one whole cycle in 200 ns.

#### Modeling of magnetic force microscopy contrast

In order to simulate the MFM frequency shift contrast at a given scanning height of 20 nm, we consider a magnetization state as our input state which was obtained by numerically solving the LLG, as explained in the previous paragraph. We increase the height of the simulation box so that the center of the last cell corresponds to the desired MFM scanning height, where all magnetic parameters are set to zero, *e.g. M*_s_ = 0, thus mimicking vacuum. The stray field **H** originating from the main magnetic body are now calculated in the large box. For the modeling of the MFM contrast, the MFM tip is assumed to be a point dipole. Note that MFM tips could be calibrated.^41,42^ Then the calibrated tip response could be integrated into the model to obtain a more quantitative comparison of the modeled with the measured MFM contrast. This was however not the focus of our work here because without a correct spatial description of anisotropy variations a quantitative agreement can never be obtained.

#### Modeling the granular distribution of defects

To simulate magnetic materials in a more realistic way, we assume a distribution of anisotropy values and direction by using the built-in Voronoi tesselation in magnum.np. To do so, we first calculate an initial Voronoi tessellation from a randomly distributed cloud of points. The cores of the Voronoi cells are then used as an input, and we iteratively smooth the corners of our Voronoi tessellation to simulate the real grains, and grain bonds of the magnetic materials used in this study. The average grain size of the Voronoi cells is about 20 nm. In the SAF layer, we assume that each magnetic layer has its own distribution. The anisotropy energy constant in each grain is pulled from a Gaussian distribution with mean = 20 kJ m^−3^ and a standard deviation of 2 kJ m^−3^. Additionally, the easy axis is pulled from a uniform distribution between −3 and +3 degrees relative to the *x*-axis of the simulation grid leading to a variation of the easy axis in the *x*–*y* plane.

#### Mechanofluidic modeling

Like in the full micromagnetic simulations before, the macrospin simulations solve the LLG but instead of discretizing the entire disk into thousands of cells and spins, the magnetization of both disks is represented by just two exchange coupled macrospins at the center of a virtual disk. This system is provided with an additional degree of freedom by letting the disk rotate according to the equations of motion derived from the conservation of angular momentum.

This simulation model is based on previous studies on spherical particles.^43,44^ It is extended to the simulation of AFC disks. The solver used in this paper applies implicit Runge–Kutta method of Radau to integrate the coupled LLG equation, angular acceleration, and the rotational velocity of the particles.

This naturally comes with a few limitations. First, the orientation of the particle is represented only by the easy axis. For a complete description of a disk rotating in 3D-space the out-of-plane axis of the particle would be required in addition to the in-plane easy axis. As a consequence, the rotation of the disk is limited to the plane of the disk and furthermore it is required that the normal vector of the disk stays perpendicular to the axis of the applied magnetic field at all times. This gives further importance to aligning the particles before applying and AC-field. After alignment the shape anisotropy will keep the magnetic spins in-plane and thus only allows for in-plane rotation. Lastly, the demagnetizing field is omitted in the effective field.

For the conservation of angular momentum we define relevant torques and angular momenta. The *z*-component of the viscous torque *τ*_visc_ is given by:2
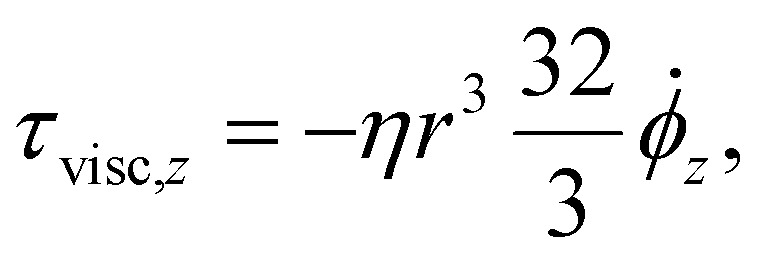
where *η* denotes the dynamic viscosity of the carrier fluid, *r* is the radius of the disk and the factor 
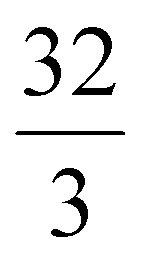
 represents the rotational friction for a disk with zero thickness and no-slip condition.^45^*φ̇*_*z*_ is the angular velocity of the rotating disk about the *z*-axis. The *z*-component of the angular momentum of a disk **L**_inert_ is given by:3
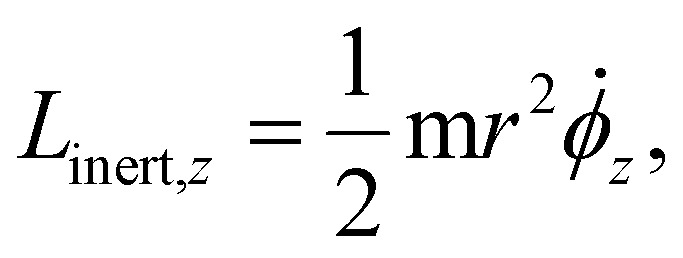
where *φ̇*_*z*_ is again the angular velocity of the particle and the other terms represent the moment of inertia of a disk spinning about its axis of rotational symmetry that corresponds in this case to the *z*-axis. 
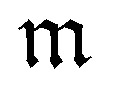
 denotes the mass of the particle. The other components of viscous torque and angular momentum are set to zero.

The magnetic contributions, namely the spin angular momentum **L**_spin_ and the torque *τ*_mag_ exerted by the external field **H**_ext_, stay the same as with the spherical particles, since they are not influenced by the change of shape:4
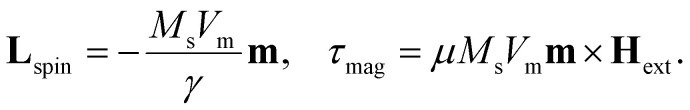


Here, *M*_s_ denotes the saturation magnetization, and *V*_m_ is the total volume of the magnetic material. The conservation of angular momentum requires that:

5 **L̇**_spin_ + **L̇**_inert_ = *τ*_mag_ + *τ*_visc_.

Combining these equations into a self-consistent solution and rearranging the terms to receive an update scheme for the rotation of the particle about its symmetry axis yields:6
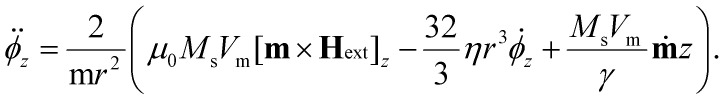


The derivative of the angular momentum of the spin is the solution to the LLG. In the case of the macrospin model, the effective field for the LLG consists of the anisotropy field, the external field and an antiferromagnetic exchange field. For this simplified model the demagnetization field is omitted. Since the SAF-MDPs are not superparamagnetic but thermally stable on the simulated time scale, thermal activation is not considered. The effect of temperature is accounted for by using material parameters at room temperature.

#### Polystyrene sphere self-assembly

Close-packed arrays of magnetic disks were fabricated using nanosphere lithography. At the beginning of this process, the wafers with magnetic thin film multilayers were treated in soft nitrogen-oxygen plasma for 5 min in order to remove contaminations and to enhance their wettability. Shortly after the plasma treatment, the wafers were submerged under surface of distilled water inside 15 cm Petri dish, where self-assembly process was carried out. Monodisperse aqueous suspension of non-functionalized and uncrosslinked polystyrene (PS) particles with average diameter of 658 nm (standard deviation of 15 nm) from MicroParticles GmbH Berlin was diluted by mixing with equal volume of ethanol (99.5% pure). The suspension was applied to the surface of the water by glass Pasteur pipette with a rate of approximately 1 μl s^−1^. When the entire surface of the water in the Petri dish has been filled with PS particles, 2D crystallization took place spontaneously leading to a highly ordered hexagonal close-packed monolayer of the particles. The monolayers were deposited on the magnetic multilayers by slow water evaporation at room temperature. Next, RF-plasma etching was used (MiniFlecto, Plasma Technology GmbH, Herrenberg, Germany), resulting in the decrease of the spheres size, but maintaining their original positions and arrangement. The plasma process was performed in oxygen and argon atmosphere under pressure of 0.15 mbar and temperature of 30 °C. During the process, a continuous flow of oxygen and argon was applied to 2 sccm and 1 sccm, respectively. After 510 s of the etching, the PS particles diameter decreased to 500 nm, which was confirmed by inspection under a scanning electron microscope.

#### Suspension fabrication and characterization

The sample concentration and elemental composition of the SAF MDPs in water was analyzed *via* inductively coupled plasma mass spectrometry (ICP-MS). Samples with a volume of 50 μL were digested in quartz tubes using a mixture of hydrochloric acid (37%, Normatom, VWR) and nitric acid (69%, Normatom, VWR) in a pressurized microwave system (TurboWAVE, MLS GmbH, Germany) at 230° Cand 120 bar for 19 minutes. After digestion, the samples were transferred to 50 ml Falcon tubes and diluted with ultrapure water. Analysis for Al, Co, Zr, and Sm was conducted using a 7900 single-quadrupole ICP-MS (Agilent Technologies, CA). Isotopes ^27^Al were measured in No-gas mode, while ^59^Co, ^90^Zr, and ^147^Sm were determined in He-collision mode. Non-spectral interferences were corrected using an internal standard solution containing Li (for Al correction) and Rh (for all other elements), which was mixed online with the sample. Calibration was performed with certified element standards (Inorganic ventures) diluted in the same acid matrix as the samples.

#### High-resolution magnetic force microscopy with in-plane fields

Performing magnetic force microscopy with high spatial resolution with negligible perturbation of the micromagnetic state of the SAF-MPDs still attached to the wafer remains a challenging experiment, requiring both specialized MFM instrumentation as well as operation modes and data processing (see chapter 4 in ref. 29).

Here we used a home-built magnetic force microscope operating in vacuum, using an SS-ISC cantilever from Team Nanotech without any coating. The tip was made sensitive to magnetic fields by sputter-deposition of a Ta(2 nm)/Co(6 nm)/Ta(4 nm) seed, magnetic and oxidation protection layer system. The thicknesses are nominal thicknesses obtained for a substrate placed perpendicular to the incoming sputtered particles. To achieve a high quality factor during vacuum operation, the cantilever base was masked^30^ to avoid coating the parts of the cantilever near the chip experiencing the highest strain upon cantilever deflection. The free resonance frequency *f*_0_ = 55.09897 kHz and quality factor *Q* = 237′192 were then found by sweeping the excitation frequency through the cantilever resonance. The cantilever stiffness *c* = 1.12 N m^−1^ is obtained from the known materials constants of silicon and the cantilever's length and width.^30^ The relatively low cantilever stiffness and high quality factor then provide a measurement sensitivity (for the chosen oscillation amplitude of *A*_rms_ = 5 nm) of 
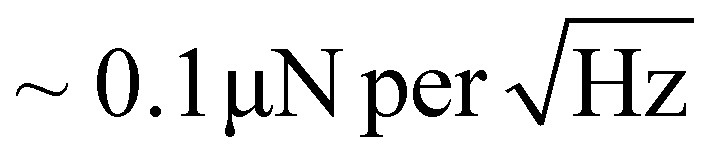
 that is about a factor of 40 above that typically obtained with MFM instruments operated with conventional cantilevers under ambient conditions.^30^ This enhanced measurement sensitivity permits the use of ultra-low magnetic moment tips which have only a small stray field and thus do not noticeably influence the micromagnetic state of the sample such that the true micromagnetic state of the SAF-MDPs can be observed.

The cantilever was driven on resonance using a phase-locked loop with an oscillation amplitude kept constant at *A*_rms_ = 5 nm. The measured quantity then is the shift of the resonance frequency away from the free cantilever resonance frequency. The MFM data acquisition was performed with the tip scanning parallel to the average slope of the sample. The distance between the tip and the nearly flat tops of the SAF-MDP islands was kept constant in average using our capacitive frequency modulated distance control operation mode.^46^

Generally forces of different physical nature are simultaneously acting on the tip of a scanning (or magnetic) force microscope and thus contribute to the measured contrast. Sophisticated differential imaging techniques were hence applied^29,30^ to disentangle the different contrast contributions and ultimately obtain the data shown in Fig. 3b–i.

In-plane fields between ±40 mT were applied to the sample by means of a permanent magnet position with an in-vacuum piezo motor linear actuator. The sign of the field was set by a rotation of the magnet such that its north or south pole was facing the sample.

#### Hyperthermia apparatus and experiments

The hyperthermia device consists of a transmission coil build of a copper hollow-conductor generating a field of 40 mT at 254 A current. In order to generate such high ac currents an oszillating circuit is used. This circuit provides reflective free impedance matching to the 50 Ω desired load for the high frequency amplifier. As frequency 305 kHz was chosen. At full field strength the voltage on the coil reach values above 700 V.

## Author contributions

H. J. H., D. S., and I. K. H. conceived the project. S. S. deposited the magnetic multilayer films, and performed the magnetometry experiments and data analysis. The polystyrene sphere self-assembly and successive oxygen plasma etching to reduce the PS sphere diameters was performed by M. K., while S. S. performed the successive ion etching to pattern the SAF-MDP islands and successively release these from the wafer support to form the SAF-MDP suspensions. The MFM and data acquisition and processing was performed by H. J. H. and R. P.-P. The micromagnetic and microfluidic modeling was performed by S. H., S. K., H. J. H., and D. S. The hyperthermia apparatus was designed and setup by H. W., J. A., and M. G., while the corresponding experiments and data analysis were performed by H. W., S. S., H. J. H, and M. G. The manuscript was conceived by H. J. H., D. S., and I. K. H., and all authors discussed and contributed to the final manuscript.

## Conflicts of interest

H.J.H and D.S. declare inventorship on a patent application related to synthetic antiferromagnetic disk nanoparticles.

## Supplementary Material

BM-013-D5BM00739A-s001

BM-013-D5BM00739A-s002

BM-013-D5BM00739A-s003

## Data Availability

The data that support the findings of this study are available from the corresponding author upon reasonable request. Supplementary information is available. See DOI: https://doi.org/10.1039/d5bm00739a.
