# Supplementary figures and images for: Approaching the physical limits of specific absorption rate for synthetic antiferromagnetic nanodisks in hyperthermia applications
Source: Biomater Sci. 2025 Sep 9;13(22):6285–97. doi: 10.1039/d5bm00739a (PMC12505462; doi:10.1039/d5bm00739a)

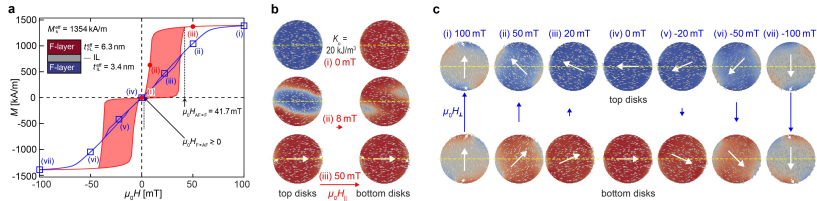

Supplement: BM-013-D5BM00739A-s001 [file BM-013-D5BM00739A-s001.pdf]
