# Supplementary material for: Approaching the physical limits of specific absorption rate for synthetic antiferromagnetic nanodisks in hyperthermia applications
Source: Biomater Sci. 2025 Sep 9;13(22):6285–97. doi: 10.1039/d5bm00739a (PMC12505462; doi:10.1039/d5bm00739a)

Supplementary Information (SI) for Biomaterials Science.
This journal is © The Royal Society of Chemistry 2025

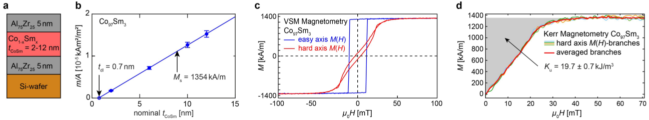

Supplement: BM-013-D5BM00739A-s002 [file BM-013-D5BM00739A-s002.pdf]
