# Supplementary material for: Approaching the physical limits of specific absorption rate for synthetic antiferromagnetic nanodisks in hyperthermia applications
Source: Biomater Sci. 2025 Sep 9;13(22):6285–97. doi: 10.1039/d5bm00739a (PMC12505462; doi:10.1039/d5bm00739a)
Supplement: BM-013-D5BM00739A-s003 [file BM-013-D5BM00739A-s003.pdf]

Supplementary Information (SI) for Biomaterials Science.
This journal is © The Royal Society of Chemistry 2025

Approaching the Physical Limits of Specific Absorption Rate in Hyperthermia Applications

S. Scheibler
*Magnetic & Functional Thin Films Laboratory, Empa,
Swiss Federal Laboratories for Materials Science and Technology,
Ueberlandstrasse 129, 8600 Dübendorf, Switzerland
Nanoparticle Systems Engineering Laboratory, Institute of Energy and Process Engineering (IEPE),
Department of Mechanical and Process Engineering (D-MAVT),
ETH Zurich, Sonneggstrasse 3, 8092 Zurich, Switzerland and
Particles-Biology Interactions Laboratory, Empa, Swiss Federal Laboratories for Materials Science and Technology,
Ueberlandstrasse 129, 8600 Dübendorf, Switzerland*

H. Wei and J. Ackers
*Fraunhofer IMTE, Fraunhofer Research Institution for Individualized and
Cell-Based Medical Engineering, Mönkhofer Weg 239a, 23562 Lübeck, Germany*

S. Helbig
*Physics of Functional Materials, Faculty of Physics,
University of Vienna, Kolingasse 14-19, 1090 Vienna, Austria and
Research Platform MMM Mathematics – Magnetism – Materials,
University of Vienna, Kolingasse 14-19, 1090 Vienna, Austria*

S. Koraltan and D. Suess
*Physics of Functional Materials, Faculty of Physic,
University of Vienna, Kolingasse 14-19, 1090 Vienna, Austria and
Research Platform MMM Mathematics – Magnetism – Materials,
University of Vienna, Kolingasse 14-19, 1090 Vienna, Austria*

R. Peremadathil-Pradeep and H. J. Hug*
*Magnetic & Functional Thin Films Laboratory, Empa,
Swiss Federal Laboratories for Materials Science and Technology,
Ueberlandstrasse 129, 8600 Dübendorf, Switzerland and
Department of Physics, University of Basel, Klingelbergstrasse 82, 4056 Basel, Switzerland*

M. Krupiński
Institute of Nuclear Physics Polish Academy of Sciences, Radzikowskiego 152, 31-342 Kraków, Poland

M. Graeser
*Institute of Medical Engineering, University of Luebeck,
Ratzeburger Allee 160, 23562 Luebeck, Germany and
Fraunhofer IMTE, Fraunhofer Research Institution for Individualized and
Cell-Based Medical Engineering, Mönkhofer Weg 239a, 23562 Lübeck, Germany*

I. K. Herrmann
*Nanoparticle Systems Engineering Laboratory, Institute of Energy and Process Engineering (IEPE),
Department of Mechanical and Process Engineering (D-MAVT),
ETH Zurich, Sonneggstrasse 3, 8092 Zurich, Switzerland
Particles-Biology Interactions Laboratory, Empa, Swiss Federal Laboratories for Materials Science and Technology,
Ueberlandstrasse 129, 8600 Dübendorf, Switzerland and
Ingenuity Lab, Balgrist University Hospital and University of Zurich, Forchstrasse 340,8008 Zürich, Switzerland*

* hans-josef.hug@empa.ch

S1 Hysteresis Loss of SPIONs.

Due to their effective magnetic anisotropy K, arising from crystalline and/or shape anisotropy, SPIONs exhibit an $M(H)$-loop with a finite hysteretic loss area A that increases with the frequency f of the applied AMF. This relationship is elucidated by Sharrock's equation [1], which demonstrates how the coercive field H_c increases as the duration of the experimental field pulse t_0 decreases, with t_0 being inversely proportional to f as

$$H_c(t_0) = H_a \left\{ 1 - \left[\frac{k_B T}{KV} \ln \left(\frac{t_0}{\ln(2)\tau_0} \right) \right]^n \right\} , \tag{S1}$$

where T is the temperature, $\tau_0 = 1/f_0 \approx 10^{-13} - 10^{-9}$ is one over the attempt frequency and $0.5 < n < 0.7$ depending on the angle of the applied field and the anisotropy axis, and $H_a = 2K/(\mu_0 M_s)$ is the anisotropy field, with K being the effective uniaxial anisotropy of the particle and M_s its saturation magnetization.

The hysteretic power loss per volume can be estimated [2] from linear response theory (LRT) as

$$P/V = f \cdot A = \mu_0 \pi H^2 f \chi'' , \tag{S2}$$

where A is the area enclosed by the $M(H)$-loop, and with the imaginary part of the susceptiblity $\chi'' = \chi_0 \cdot [2\pi f\tau/(1+2\pi f\tau)^2]$ describing the de-phasing of the magnetic moment of the magnetic nanoparticles (MNPs) with respect to the AMF. $\chi_0 = \frac{\mu_0 M_s^2 V}{3k_B T}$ is the static susceptibility depending on the MNP's saturation magnetization M_s and their volume V. The hysteretic loss area A of the $M(H)$-loop reaches a maximum for a frequency f related to the total relaxation time τ as $2\pi f\tau = 1$. The total relaxation time is given by $\tau^{-1} = \tau_N^{-1} + \tau_B^{-1}$ where $\tau_N = \tau_0 \cdot \exp\left(\frac{KV}{k_B T}\right)$ with a pre-exponential factor $\tau_0 \approx 10^{-13} - 10^{-9}$ s is the Néel relaxation time, and $\tau_B = \frac{3V_H \eta}{k_B T}$ is the Brown relaxation time arising from the MNPs rotation in the liquid with the viscosity η and a hydrodynamic MNP volume $V_H > V$.

The heating efficacy of magnetic nanoparticles is typically given by the specific loss parameter SLP in Watts per gramm magnetic material as

$$\mathrm{SLP} := \frac{\mathrm{P}}{\rho} = \frac{\mathrm{Af}}{\rho} , \tag{S3}$$

where ρ is the density of the used magnetic material.

Note that both relaxation mechanisms contribute to the total power loss but the latter remains directly related to the hysteretic loss area A of the $M(H)$-loop occurring at the frequency f of the AMF [3]. LRT however overestimates the power loss. For this reason, and also to incorporate inter-particle interactions, Ruta et al. [4] developed a kinetic Monte Carlo model to calculate the SLP for a suspension of SPIONs with a radii $r = 7$ to 20 nm using a saturation magnetization of 400 kA/m and an anisotropy $K = 30$ kJ/m^3 typical for magnetite. A power loss of about 300 W/g was obtained for an AMF frequency $f = 100$ kHz and a field amplitude $\mu_0 H = 30$ mT. Note that for non-interacting MNPs SLP values up to 670 W/g have been obtained for an optimized particle diameter $D = 13.8$ nm. It is noteworthy that an ensemble of MNP having a finite size distribution will exhibit a markedly reduced SLP value. This reduction is primarily because the majority of MNPs possess diameters that deviate from the optimum size required for a specific frequency and field intensity of the alternating magnetic field (AMF).

S2 Limited Hysteretic Loss of SAF-MDPs prior to our Work

To date, all SAF-MDPs with an in-plane magnetization showed an almost linear $M(H)$-loop without any hysteresis reminiscent to a Stoner-Wohlfarth [5] like hard axis magnetization process. Hu et al. [6, 7] attributed this to a spin-flop process, where the initially antiparallel magnetization directions of the two FLs flip away from $\mathbf{e}_{K_\mathrm{u}}$ for fields $H \parallel \mathbf{e}_{K_\mathrm{u}} > H_\mathrm{sf}$, where H_sf is the spin-flop field, and gradually scissor towards $\mathbf{e}_{K_\mathrm{u}}$ for increasing fields, leading to an almost linear and hysteresis-free $M(H)$-loop. Hence SAF-MDP with in-plane have so far only been designed for applications like separation of biomolecules or cell manipulation and sorting [8] but because of the lack of a magnetic hysteresis are not suited for hyperthermia applications.

Vemulkar et al. [9] fabricated SAF-MDPs with perpendicular magnetization states. In liquid, these particles align their hard axis to the field for fields up to about 140 to 300 mT, resulting in a linear $M(H)$-loop without hysteresis. At higher fields, the SAF-MDPs align their easy axis to the applied field, accompanied by a small hysteretic loss area. Consequently, the total hysteretic losses remain small, rendering these particles unsuitable for hyperthermia applications. Consequently, subsequent research [10] focused on the mechanical response of such perpendicular SAF-MDPs in liquid for magnetomechanical cancer cell destruction [11, 12]. However, disk-shaped nanoparticles comprised of only one ferromagnetic layer (using inexpensive magnetic materials) forming a magnetic vortex state have also been used for magnetomechanical cancer cell destruction [13–16].

SAF-MDPs with larger easy axis hysteretic losses have recently been reported by Li et al. [17] for disk diameters of 1.8 μm and 120 nm. However, the switching fields were too high for hyperthermia applications and the shape of the $M(H)$-loop is rounded by wide switching field distributions typically occurring patterned films with perpendicular magnetization [18–23].

S3 Micromagnetic Modeling based System Design

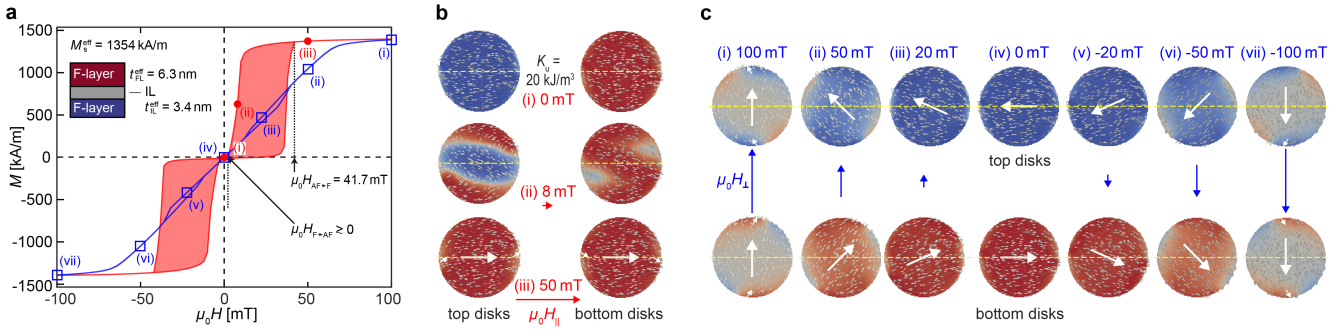

FIG. S1. **Micromagnetic modeling based design of an in-plane SAF-MDP with 20 kJ/m^3 uniaxial anisotropy a** easy (red curve) and hard axis (blue curve) $M(H)$-loop with large and vanishing hysteretic loss. The top left inset displays the SAF layer structure. **b** micromagnetic states of the upper and lower F layer for fields of 0 mT, 8 mT, and 50 mT applied along the easy axis (dashed yellow lines). **c** micromagnetic states of the upper and lower F layer for fields of 100 mT, 50 mT, 20 mT, 0 mT, -20 mT, -50 mT and -100 mT revealing the hysteresis-free rotation of the magnetic moments for fields applied perpendicular to the easy axis (yellow lines).

Fig. S1**a** illustrates the hysteretic easy axis $M(H)$-loop (red curve) together with the (essentially) non-hysteretic hard axis $M(H)$-loop (blue curve) obtained from our micromagnetic modelling for an SAF-MDP with a diameter of

500 nm, an effective FL thickness of $t_{\mathrm{FL}}^{\mathrm{eff}} = 6.3\,\mathrm{nm}$, an effective interlayer thickness of $t_{\mathrm{IL}}^{\mathrm{eff}} = 3.4\,\mathrm{nm}$, a uniaxial in-plane anisotropy of $20\,\mathrm{kJ/m^3}$, and an effective saturation magnetization $M_{\mathrm{s}}^{\mathrm{eff}} = 1354\,\mathrm{kA/m}$ (here the effective thicknesses and saturation magnetization describes the quantities as found for our experimental SAF-MDP system). Fig. S1**b** displays the micromagnetic states for fields of 0 mT, 8 mT and 50 mT, demonstrating that the substantial uniaxial anisotropy present here (along the dashed yellow lines) can effectively suppress the spin-flop process, thereby enabling a hysteretic easy axis magnetization loop. Note also that an anisotropy much higher than $20\,\mathrm{kJ/m^3}$ is unfavorable, as it would increase the $H_{\mathrm{AF}\to\mathrm{F}} = 41.7\,\mathrm{mT}$ switching field and thus the required AMF amplitude, and prevent the SAF-MDP from returning to its AF ground state at zero field. Additionally, it would lead to a reduction of the domain wall thickness from the present $\delta_{\mathrm{dw}} = \pi\sqrt{\frac{A}{K_{\mathrm{u}}}} = 82\,\mathrm{nm}$, where $A \approx 15\,\mathrm{pJ/m}$ is the exchange stiffness assumed for a typical FL material. For a real SAF-MDP with defects, this could lead to undesirable multi-domain states and increased domain wall pinning, broadening the transition between AF and F-states as for example observed for perpendicular SAF-MDP [17]. Fig. S1**c** then illustrates the rotatory, (essentially) hysteresis-free $M(H)$-loop (blue curve) obtained for fields applied perpendicular to the easy axis (dashed yellow lines).

S4 Fabrication of a Ferromagnetic Layer with a large in-plane Anisotropy

While interface anisotropy readily generates large perpendicular anisotropies in magnetic multilayers, achieving substantial in-plane anisotropies requires alternative strategies. An in-plane uniaxial anisotropy of about $25\,\mathrm{kJ/m^3}$ could for example be achieved for a 6 nm-thick CoFeB layer sputter-deposited onto a 15 nm-thick Ta seed layer fabricated by oblique sputter-deposition with an angle of 60° [24]. However, the obtained anisotropy is of interface type and hence decays with the inverse thickness of the FL making it challenging to obtain an equal anisotropy in both FLs of an SAF-MDP.

Here, we thus perform an in-field sputter-deposition of amorphous $\mathrm{Co}_{1-x}\mathrm{Sm}_x$ layers for which anisotropies up to about $200\,\mathrm{kJ/m^3}$ were obtained for $x = 0.2$ [25]. For the $20\,\mathrm{kJ/m^3}$ required according to our micromagnetic modeling work, much smaller concentrations of Sm were tested and an optimal concentration of 3% was determined. Further, to promote the amorphous growth, $\mathrm{Al}_{75}\mathrm{Zr}_{25}$ layers were used for the 5 nm-thick seed layer, 2 nm thick interlayer and for the 5 nm-thick oxidation protection layer.

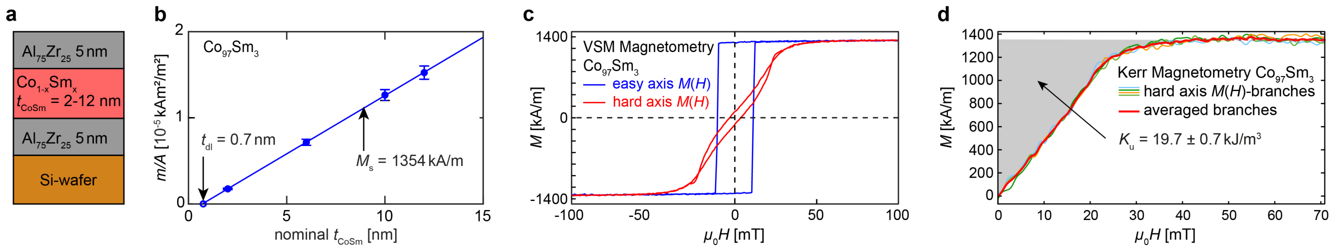

FIG. S2. **Materials selection and SAF-MDP fabrication.** **a** multilayer system containing a single $\mathrm{Co}_{1-x}\mathrm{Sm}_x$ layer as used for the determination of the dead layer thickness, and the evaluation of the anisotropy. **b** magnetic moment of $\mathrm{Co}_{97}\mathrm{Sm}_3$ layers as a function of the layer thickness as used for the determination of the dead layer thickness, and saturation magnetization. **c** easy (blue curve) and hard (red) axis VSM data. **d** Kerr magnetometry results used for the determination of the uniaxial anisotropy.

To investigate the total thickness of magnetic dead layers potentially forming at the $\mathrm{Co}_{1-x}\mathrm{Sm}_x/\mathrm{Al}_{75}\mathrm{Zr}_{25}$ interfaces,

a series of samples with F-layer thicknesses t_{CoSm} between 2 and 12 nm were fabricated and analyzed by VSM. Fig. S2**b** displays the magnetic moment as a function of the magnetic layer thickness t_{CoSm} together with a straight line fit. Its interception with the thickness axis then corresponds to the thickness of the total magnetic dead layer ($t_{\mathrm{dl}} = 0.7\,\mathrm{nm}$) forming at the interfaces to the adjacent $Al_{75}Zr_{25}$ layers resulting in an effective thickness of the FL hence $t_{\mathrm{FL}}^{\mathrm{eff}} = 7\,\mathrm{nm} - t_{\mathrm{dl}} = 6.3$ nm, while the slope of 1354 kA/m corresponds to the effective saturation magnetization $M_{\mathrm{s}}^{\mathrm{eff}}$. The dead layer thickness also enhances the interlayer thickness from the nominally 2 nm to 3.4 nm. $M(H)$-loops for a nominally 7 nm-thick $Co_{97}Sm_3$ film were acquired by VSM with the easy (blue curve) and hard (red) axis loops diplayed in Fig. S2**c**. The hard axis $M(H)$-loop deviates from the expected ideal linear and hysteresis free $M(H)$-loop which can be attributed to hysteretic processes occurring near the sample edges. A perfectly linear and hysteresis free hard axis $M(H)$-loop could however be obtaind by Kerr magnetometery (Fig. S2**d**) which permits localized measurements in the center of a larger sample with a fine adjustment of the angle of the applied field but cannot obtain a value for M_{s}. Using the $M_{\mathrm{s}}^{\mathrm{eff}}$ from the VSM measurement, an anisotropy $K_{\mathrm{u}} = (19.7 \pm 0.7)\,\mathrm{kJ/m^3}$ close to the $20\,\mathrm{kJ/m^3}$ used for the micromagnetic modeling (Fig. S1**a**) was determined from the area enclosed between the magnetization axis and the averaged four 0-to-M_{s}-branches of the $M(H)$ Kerr magnetometry data.

[1] M. P. Sharrock, Time dependence of switching fields in magnetic recording media (invited), Journal of Applied Physics **76**, 6413 (1994).

[2] R. Hergt and S. Dutz, Magnetic particle hyperthermia—biophysical limitations of a visionary tumour therapy, Journal Of Magnetism And Magnetic Materials **311**, 187 (2007).

[3] S. Helbig, C. Abert, P. A. Sánchez, S. S. Kantorovich, and D. Suess, Self-consistent solution of magnetic and friction energy losses of a magnetic nanoparticle, Physical Review B **107**, 054416 (2023), 2204.14106.

[4] S. Ruta, R. Chantrell, and O. Hovorka, Unified model of hyperthermia via hysteresis heating in systems of interacting magnetic nanoparticles., Scientific Reports **5**, 9090 (2015).

[5] E. C. Stoner and E. P. Wohlfarth, A mechanism of magnetic hysteresis in heterogeneous alloys, Philosophical Transactions of the Royal Society of London. Series A, Mathematical and Physical Sciences **240**, 599 (1948).

[6] W. Hu, R. J. Wilson, A. Koh, A. Fu, A. Z. Faranesh, C. M. Earhart, S. J. Osterfeld, S.-J. Han, L. Xu, S. Guccione, R. Sinclair, and S. X. Wang, High-Moment Antiferromagnetic Nanoparticles with Tunable Magnetic Properties, Advanced Materials **20**, 1479 (2008).

[7] W. Hu, R. J. Wilson, C. M. Earhart, A. L. Koh, R. Sinclair, and S. X. Wang, Synthetic antiferromagnetic nanoparticles with tunable susceptibilities, Journal Of Applied Physics **105**, 07B508 (2009).

[8] M. Zhang, C. M. Earhart, C. Ooi, R. J. Wilson, M. Tang, and S. X. Wang, Functionalization of high-moment magnetic nanodisks for cell manipulation and separation, Nano Research **6**, 745 (2013).

[9] T. Vemulkar, R. Mansell, D. C. M. C. Petit, R. P. Cowburn, and M. S. Lesniak, Highly tunable perpendicularly magnetized synthetic antiferromagnets for biotechnology applications, Applied Physics Letters **107**, 012403 (2015).

[10] T. Vemulkar, E. N. Welbourne, R. Mansell, D. C. M. C. Petit, and R. P. Cowburn, The mechanical response in a fluid of synthetic antiferromagnetic and ferrimagnetic microdiscs with perpendicular magnetic anisotropy, Applied Physics Letters **110**, 042402 (2017).

[11] Y. Cheng, M. E. Muroski, D. C. Petit, R. Mansell, T. Vemulkar, R. A. Morshed, Y. Han, I. V. Balyasnikova, C. M. Horbinski, X. Huang, L. Zhang, R. P. Cowburn, and M. S. Lesniak, Rotating magnetic field induced oscillation of magnetic

particles for in vivo mechanical destruction of malignant glioma, Journal of Controlled Release **223**, 75 (2016).

[12] R. Mansell, T. Vemulkar, D. C. M. C. Petit, Y. Cheng, J. Murphy, M. S. Lesniak, and R. P. Cowburn, Magnetic particles with perpendicular anisotropy for mechanical cancer cell destruction, Scientific Reports **7**, 4257 (2017).

[13] D.-H. Kim, E. A. Rozhkova, I. V. Ulasov, S. D. Bader, T. Rajh, M. S. Lesniak, and V. Novosad, Biofunctionalized magnetic-vortex microdiscs for targeted cancer-cell destruction, Nature Materials **9**, 165 (2010).

[14] S. Leulmi, X. Chauchet, M. Morcrette, G. Ortiz, H. Joisten, P. Sabon, T. Livache, Y. Hou, M. Carrière, S. Lequien, and B. Dieny, Triggering the apoptosis of targeted human renal cancer cells by the vibration of anisotropic magnetic particles attached to the cell membrane, Nanoscale **7**, 15904 (2015).

[15] M. Goiriena-Goikoetxea, D. Muñoz, I. Orue, M. L. Fernández-Gubieda, J. Bokor, A. Muela, and A. García-Arribas, Disk-shaped magnetic particles for cancer therapy, Applied Physics Reviews **7**, 011306 (2020).

[16] C. Naud, C. Thébault, M. Carrière, Y. Hou, R. Morel, F. Berger, B. Diény, and H. Joisten, Cancer treatment by magneto-mechanical effect of particles, a review, Nanoscale Advances **2**, 3632 (2020).

[17] J. Li, P. v. Nieuwkerk, M. A. Verschuuren, B. Koopmans, and R. Lavrijsen, Substrate conformal imprint fabrication process of synthetic antiferromagnetic nanoplatelets, Applied Physics Letters **121**, 182407 (2022), 2206.15320.

[18] S. Adhikari, J. Li, Y. Wang, L. Ruijs, J. Liu, B. Koopmans, M. Orrit, and R. Lavrijsen, Optical Monitoring of the Magnetization Switching of Single Synthetic-Antiferromagnetic Nanoplatelets with Perpendicular Magnetic Anisotropy, ACS Photonics **10**, 1512 (2023).

[19] J.-P. Jamet, S. Lemerle, P. Meyer, J. Ferré, B. Bartenlian, N. Bardou, C. Chappert, P. Veillet, F. Rousseaux, D. Decanini, and H. Launois, Dynamics of the magnetization reversal in Au/Co/Au micrometer-size dot arrays, Physical Review B **57**, 14320 (1998).

[20] T. Thomson, G. Hu, and B. Terris, Intrinsic Distribution of Magnetic Anisotropy in Thin Films Probed by Patterned Nanostructures **96**, 10.1103/physrevlett.96.257204 (2006).

[21] J. M. Shaw, W. H. Rippard, S. E. Russek, T. Reith, and C. M. Falco, Origins of switching field distributions in perpendicular magnetic nanodot arrays, Journal of Applied Physics **101**, 023909 (2007).

[22] J. W. Lau, R. D. McMichael, S. H. Chung, J. O. Rantschler, V. Parekh, and D. Litvinov, Microstructural origin of switching field distribution in patterned CoPd multilayer nanodots, Applied Physics Letters **92**, 012506 (2008).

[23] B. Pfau, C. M. Günther, E. Guehrs, T. Hauet, H. Yang, L. Vinh, X. Xu, D. Yaney, R. Rick, S. Eisebitt, and O. Hellwig, Origin of magnetic switching field distribution in bit patterned media based on pre-patterned substrates, Applied Physics Letters **99**, 062502 (2011).

[24] S. Scheibler, O. Yildirim, I. Herrmann, and H. Hug, Inducing in-plane uniaxial magnetic anisotropies in amorphous CoFeB thin films, Journal of Magnetism and Magnetic Materials **585**, 171015 (2023).

[25] F. Magnus, R. Moubah, A. H. Roos, A. Kruk, V. Kapaklis, T. Hase, B. Hjörvarsson, and G. Andersson, Tunable giant magnetic anisotropy in amorphous SmCo thin films, Applied Physics Letters **102**, 10.1063/1.4802908 (2013).
